# Magnetic field sensor based on a combination of a microfiber coupler covered with magnetic fluid and a Sagnac loop

**DOI:** 10.1038/s41598-017-05199-y

**Published:** 2017-07-05

**Authors:** Fangfang Wei, Arun Kumar Mallik, Dejun Liu, Qiang Wu, Gang-Ding Peng, Gerald Farrell, Yuliya Semenova

**Affiliations:** 1 Photonics Research Centre, School of Electrical and Electronic Engineering, Dublin Institute of Technology, Kevin St, Dublin 8 Ireland; 2 Department of Mathematics, Physics and Electrical Engineering, Northumbria University, Newcastle Upon Tyne, NE1 8ST United Kingdom; 3 Photonics and Optical Communications, School of Electrical Engineering and Telecommunications, University of New South Wales, Sydney, 2052 NSW Australia

## Abstract

This paper proposes a novel magnetic field sensor based on a microfiber coupler (MFC) combined with a magnetic fluid (MF) in a Sagnac loop formed from a polarization maintaining fiber (PMF). Thanks to the small (~2.6 *μ*m) waist diameter of the MFC, the resulting interference is strongly influenced by the presence of the MF and this leads to the desirable high sensitivity of the structure to the applied magnetic field. The maximum magnetic field sensitivities of −100 pm/mT and −488 pm/mT have been experimentally demonstrated with the PMF lengths of 75 cm and 20 cm respectively in the range of magnetic field strengths from 0 to 200 mT. The dependence of the magnetic field orientation on the performance of the proposed sensor was also examined. The proposed magnetic field sensor is advantageous for applications requiring higher sensitivity over a wide magnetic field range.

## Introduction

Optical fiber magnetic field sensors using magnetic fluids (MFs) as sensing materials have aroused significant research interest due to the magneto-optical properties of MFs, such as field dependent refractive index (RI), optical birefringence and transmission^1, 2^ which have the potential to allow the development of very sensitive sensing structures. Water-based MFs are highly stable colloidal suspensions of magnetic nano-particles distributed evenly throughout the volume, which makes them easy to integrate with optical fibers. Several magnetic field sensors have been developed by combining optical devices with magnetically susceptible fluids utilizing the tunability of the magnetic fluid’s RI to measure the external magnetic field strength. For example, a sensor based on a magnetic fluid clad etched fiber Bragg grating^3^ and a sensor using tilted fiber grating interacting with magnetic fluid^4^ have been reported. It should be noted, that since interaction between the fundamental core mode and lower-order cladding modes in such structures is usually quite low, such sensors suffer from relatively low sensitivity to magnetic fields, normally less than 10 pm/mT. Zu *et al*.^5^ proposed a magnetic field sensor with a higher sensitivity of ~16.7 pm/Oe but a more complex structure based on a Sagnac interferometer incorporating a free-space MF film with the use of two collimators.

Among the variety of fiber optic structures suitable for optical sensing, microfiber couplers (MFCs) have become a topic of special interest due to their superior sensing properties compared to other fiber structures and conventional fused fiber couplers^6–8^. Microfiber based magnetic field sensors show higher sensitivity to external RI than traditional optical structures utilizing interference because their much smaller diameter means that a larger proportion of the evanescent field surrounding the microfiber can interact with the local environment. For example, Zheng *et al*.^9^ proposed an optical microfiber mode interferometer coated by a MF with a sensitivity of −29.3 pm/Oe and Deng *et al*.^10^ reported a magnetic field sensor with a sensitivity of ~162 pm/mT, composed from an asymmetric taper with a waist diameter of 45 *μ*m immersed in a magnetic fluid. However, the fabrication of such an asymmetric taper structure requires accurate manual adjustment of the fibers’ positions within the fusion splicer during the application of multiple discharges leading to poor reproducibility. Luo *et al*.^11^ reported a magnetic field sensor with an MFC surrounded with a magnetic fluid, with a sensitivity of 191.8 pm/Oe in a narrow range of weak magnetic fields of up to ~300 Oe. Several researchers^12, 13^ reported experimental and theoretical studies of fiber sensors where an MFC was combined with a Sagnac loop for sensing of refractive index or magnetic field, showing that a very high sensitivity is achievable with such structures.

In this paper we propose and examine a novel magnetic field sensing structure formed by connecting two ends of an MFC immersed in a magnetic fluid with a section of polarization maintaining fiber (PMF) to form a Sagnac loop. The introduction of a PMF in an MFC-Sagnac loop sensing structure has not been investigated before and allows for control over both the free spectral range (FSR) of the transmission spectrum and the sensitivity of the sensor by adjusting the length of the PMF section. We investigate for the first time the properties of the proposed magnetic field sensor, its hysteresis behavior and the dependence of the sensor’s performance on the applied magnetic field orientation. The proposed structure offers relative simplicity, good repeatability for fabrication and demonstrates a competitive magnetic field sensitivity of −488 pm/mT in the magnetic field range from 0 to 200 mT. The sensitivity of the proposed sensor could be further improved by optimizing the parameters of the structure using a simple analytical model also presented here.

## Results

### Proposed structure and operating principle

**Figure 1:**
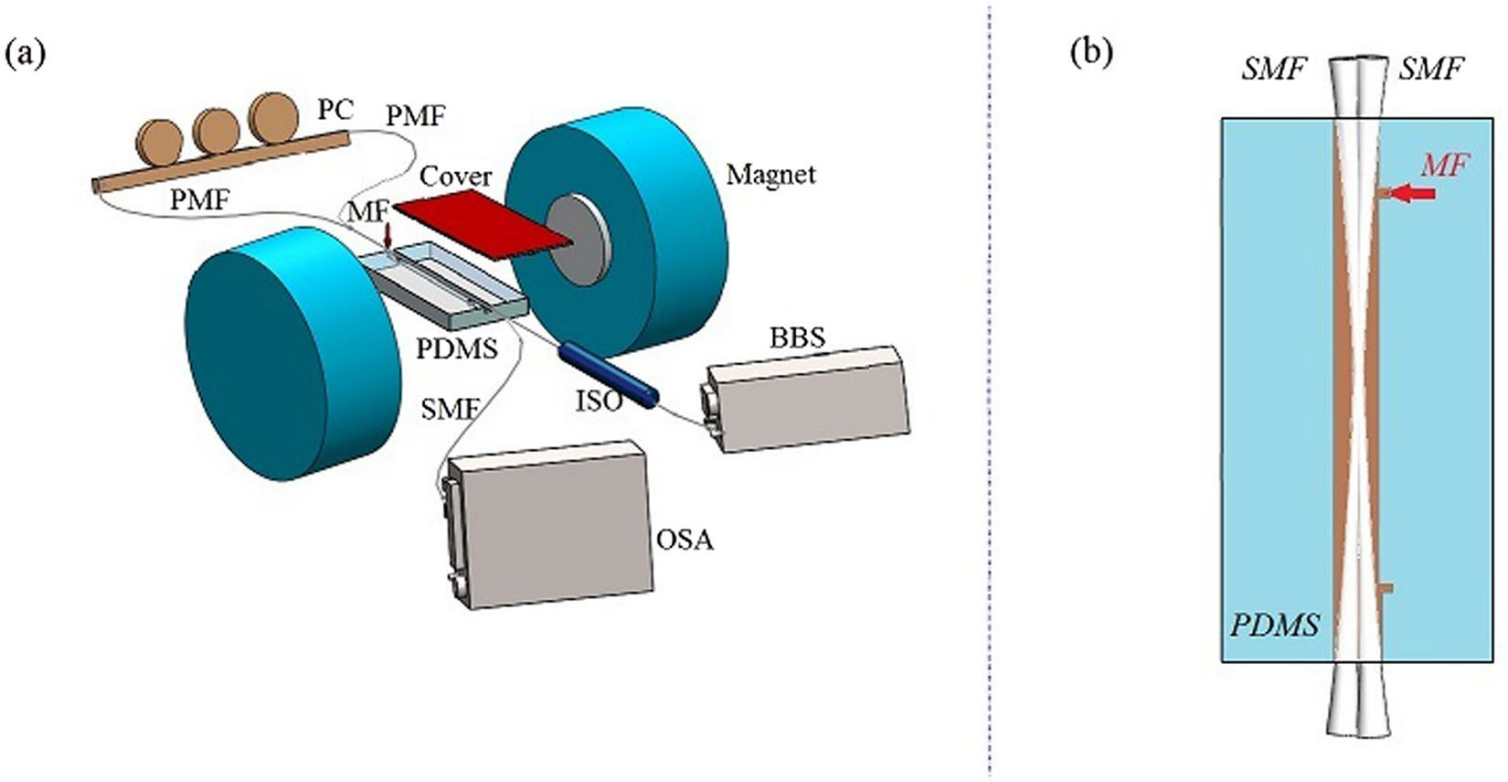
(**a**) Schematic diagram of the proposed structure and its characterization setup; (**b**) top view of the MFC encapsulated in a PDMS container with MF.

A schematic diagram of the proposed magnetic field sensor is shown in Fig. 1(a). It includes an MFC encapsulated in a polydimethylsiloxane (PDMS) container filled with MF acting as the sensor head (Fig. 1(b)). The waist diameter of the MFC is fabricated to 2.6 *μ*m. A section of polarization maintaining fiber (PMF) with a length of ~75 cm connects the two ends of the MFC at the same side to form a Sagnac loop interferometer. A broadband source (BBS) with an output spectrum from 1500 nm to 1580 nm serves as the input light source and the output spectrum is observed using an optical spectrum analyzer (OSA) with a resolution of 20 pm. A polarization controller (PC) is inserted into the loop to control the input light polarization state. An optical isolator (ISO) is inserted between the BBS and MFC to prevent backward propagating light from reaching the BBS.

The operating principle of a Sagnac loop is based on the multi-beam interference between the clockwise and counterclockwise beams in the fiber loop. The MFC itself typically has a transmission spectrum containing interference fringes, the visibility and free spectral range (FSR) of which along with the coupling ratio are strongly affected by the coupler’s waist diameter^14^. By utilizing the birefringence effect in the PMF section, it is possible to achieve a desired FSR value for the combined MFC-Sagnac structure by variations of the length of the PMF section. When magnetic field acts upon the MFC waist section covered with the MF, the overall interference spectrum changes due to the variation in the magnetic fluid’s RI. As a result, by measuring the parameters of interference spectrum, the strength of the applied magnetic field can be monitored assuming a suitable calibration has taken place.

### Theoretical analysis and simulation

In a conventional Sagnac loop structure, a standard fiber coupler is used to split the light into two beams which counter-propagate within the PMF loop. Propagating within the PMF loop the two light beams accumulate a phase difference before re-combining at the coupler and they produce interference due to the acquired phase difference. The transmission equation for a conventional coupler-based Sagnac loop is as follows^15^:

1 $$T(\lambda )={\mathrm{(1}-2r)}^{2}+4r\mathrm{(1}-r)\cdot {\sin }^{2}({\theta }_{1}+{\theta }_{2})\cdot {\cos }^{2}(\frac{-2\pi l({n}_{o}-{n}_{e})}{\lambda })$$

where *l* is the length of the PMF section, (*n*_*o*_ − *n*_*e*_) is the birefringence of the PMF, *λ* is the operating wavelength and *θ*_1_ and *θ*_2_ are the angles between light polarizations at both ends of the PMF and *r* is the coupling ratio of the fiber coupler. Light whose polarization is perpendicular to the optical axis is governed by the refractive index *n*_*o*_. Light whose polarization is in the direction of the optic axis sees an optical index *n*_*e*_. Given a fixed length of the PMF, the birefringence (*n*_*o*_ − *n*_*e*_) is constant. Standard commercial fiber couplers are designed to realize a fixed coupling ratio over a wide wavelength range (typically ~30 nm), in effect *r* is independent of wavelength over a wide range. Furthermore the *r* is independent of the RI surrounding the coupler. In our proposed configuration a conventional fiber coupler is replaced by an MFC whose coupling coefficient (*r*_*MFC*_) is both wavelength- and surrounding RI-dependent^16^:

2 $${r}_{MFC}=\frac{\pi \sqrt{{n}_{1}^{2}-{n}_{2}^{2}}}{2a{n}_{1}}{e}^{-2.3026(A+B(\frac{d}{a})+C{(\frac{d}{a})}^{2})}$$

where

$$\begin{array}{cccccccccccc}A & = & {a}_{1}+{a}_{2}V+{a}_{3}{V}^{2} & {a}_{1} & = & 2.2926 & {a}_{2} & = & -1.5910 & {a}_{3} & = & 0.1668\\ B & = & {b}_{1}+{b}_{2}V+{b}_{3}{V}^{2} & {b}_{1} & = & -0.3347 & {b}_{2} & = & 0.5321 & {b}_{3} & = & -0.0066\\ C & = & {c}_{1}+{c}_{2}V+{c}_{3}{V}^{2} & {c}_{1} & = & -0.0076 & {c}_{2} & = & -0.0028 & {c}_{3} & = & 0.0004\end{array}$$

*n*_1_ and *n*_2_ are the RIs of the silica fiber and surrounding medium, *a* is the radius of the microfiber waist, *d* is the center-to-center spacing between fibers forming the MFC, and *V* is the normalized frequency defined as $V=\frac{\mathrm{(2}\pi a)}{\lambda }\sqrt{({n}_{1}^{2}-{n}_{2}^{2})}$.

**Figure 2:**
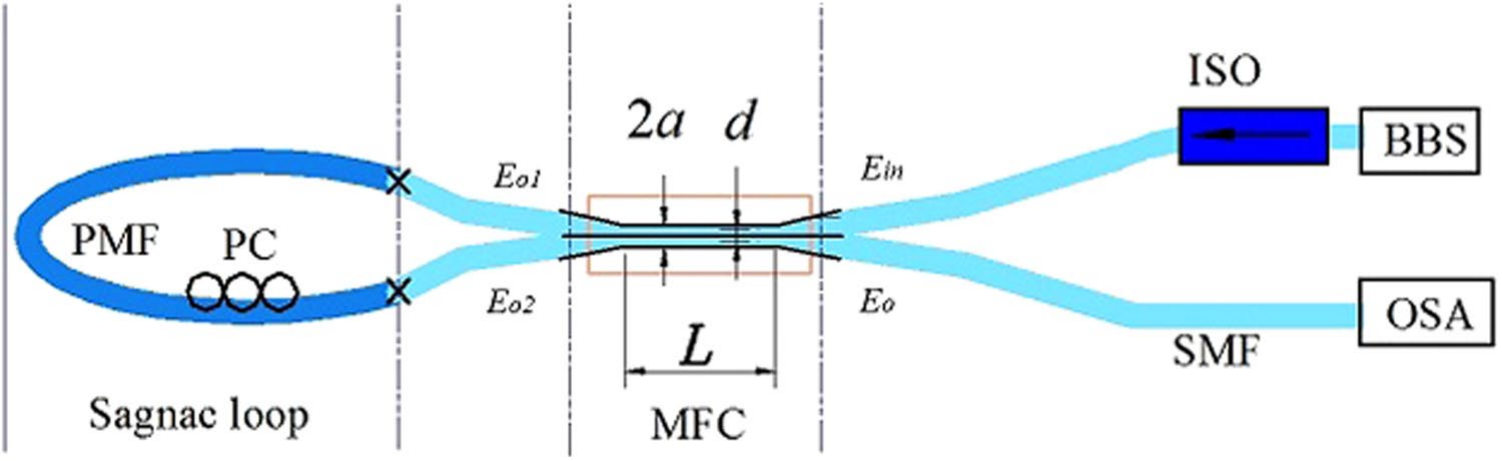
Schematic diagram of the proposed fiber structure.

Equation (2) allows for the analysis of the influence of the MFC geometric parameters and surrounding RI on its coupling coefficient in the wavelength range of interest. A schematic diagram illustrating key parameters of the MFC, such as the coupler waist diameter (2*a*), the center-to-center spacing between the fibers (*d*) and length of the waist section (*L*), is shown in Fig. 2.

Assuming input light with amplitude *E*_*in*_ enters the MFC, the output light in the through port (*E*_*o*1_) and the coupling port (*E*_*o*2_) are:

3 $${E}_{o1}={E}_{in}\,\sin ({r}_{MFC}\cdot L),\quad {E}_{o2}={E}_{in}\,\cos ({r}_{MFC}\cdot L)$$

After incorporating the PMF loop, light from both the through and coupling ports of the MFC will counter-propagate within the loop, experiencing interference upon reentering the MFC. The resulting transmission equation for proposed MFC-Sagnac fiber structure (at the output *E*_*o*_, Fig. 2) can then be written as follows:

4 $${T}_{MFC-Sagnac}(\lambda )=T(\lambda )\cdot {\cos }^{2}({r}_{MFC}\cdot L)$$

In Sagnac loop utilizing a conventional coupler, the input light *E*_*in*_ splits into *E*_01_ and *E*_02_ thus^15^:

5 $${E}_{01}=\sqrt{r}{E}_{in},\quad {E}_{02}=\sqrt{1-r}{E}_{in}$$

Comparing Eqs (3) and (5), the coupling ratio of the proposed MFC- Sagnac loop can be expressed as:

6 $${r}_{MFC-Sagnac}={\sin }^{2}({r}_{MFC}\cdot L)$$

Therefore the transmission equation for the proposed structure can be written as:

7 $$\begin{array}{rcl}{T}_{MFC-Sagnac}(\lambda ) & = & [{\mathrm{(1}-2{r}_{MFC-Sagnac})}^{2}+4{r}_{MFC-Sagnac}\mathrm{(1}-{r}_{MFC-Sagnac})\\  &  & \cdot {\sin }^{2}({\theta }_{1}+{\theta }_{2})\cdot {\cos }^{2}(\frac{-2\pi l({n}_{o}-{n}_{e})}{\lambda })]\cdot {\cos }^{2}({r}_{MFC}\cdot L)\end{array}$$

Analysis of Eqs (2) and (7) shows that if the PMF loop parameters are fixed, the transmission spectrum of the proposed structure will only be influenced by the coupling coefficient of the MFC, which in turn is determined by its geometry and the surrounding RI.

**Figure 3:**
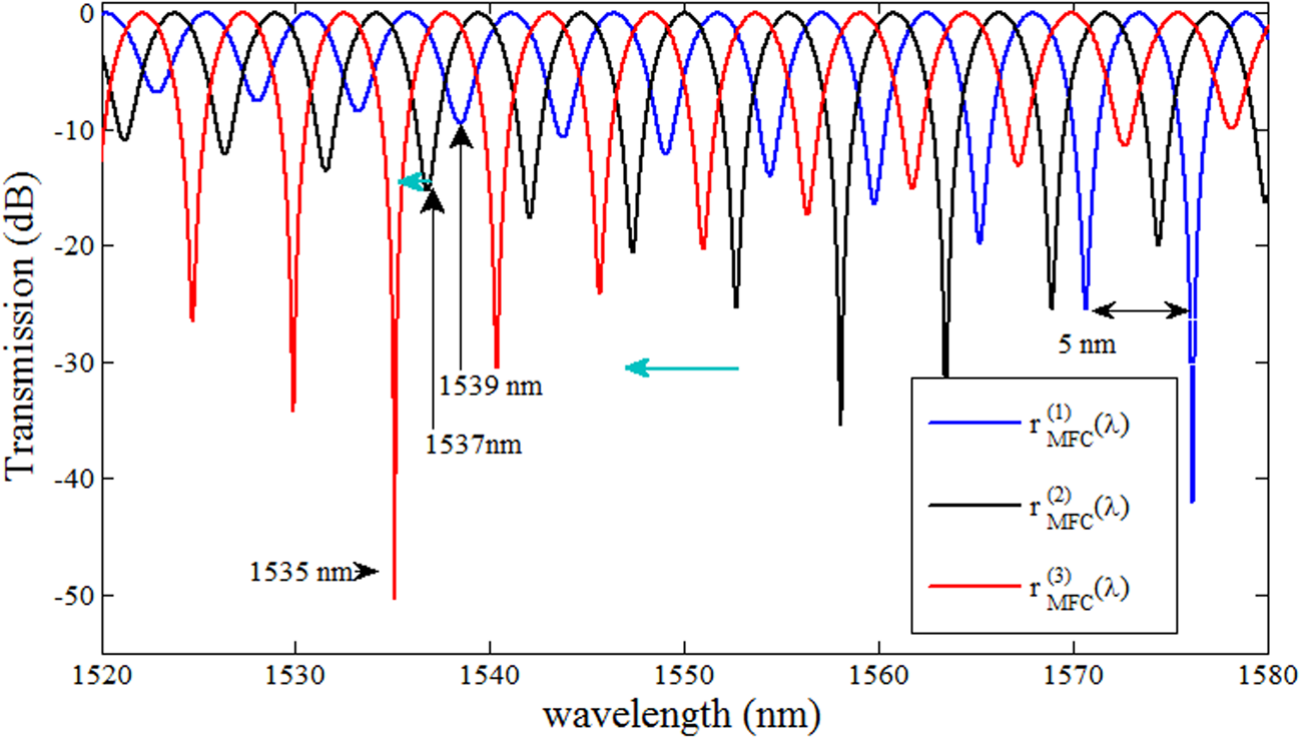
Simulated transmission of the proposed structure for different values of the MFC coupling ratio.

Figure 3 shows the results of simulations according to eq. (7) for three different values of the coupling coefficient corresponding to an MFC with the same geometry immersed in liquids with RIs of 1.33, 1.35 and 1.37 resulting in three different values of wavelength dependent coupling ratio: ${r}_{MFC}^{1}(\lambda ),{r}_{MFC}^{2}(\lambda )$ and ${r}_{MFC}^{3}(\lambda )$ respectively. The diameter 2*a* of the MFC used in the simulation is 2.6 *μm*, the coupling length *L* is 2 mm, the center-to-center spacing *d* is 1.26 *μm*, and the length of the PMF *l* is 0.75 m. As can be seen from the figure increasing the MFC coupling ratio $\{{r}_{MFC}^{1}(\lambda ) < {r}_{MFC}^{2}(\lambda ) < {r}_{MFC}^{3}(\lambda )\}$ causes a blue shift of the spectral dip with the highest extinction ratio. Measurement of the spectral position of this dip can be related to the value of *r*_*MFC*_(*λ*) which is influenced by the RI surrounding the MFC waist.

As can be seen from the above analysis, unlike conventional couplers typically used in a Sagnac loop, the coupling coefficient of the MFC strongly depends on the changes in the surrounding RI due to the large proportion of the evanescent field which extends into the medium in the vicinity of the tapered waist section. This strongly affects the interference conditions for the light entering the two ports of the PMF loop. In the proposed fiber sensor structure, the MFC, whose tapered waist is coated with a layer of a magnetic fluid, acts as a magnetic field sensing element. An external magnetic field applied to the MFC waist region forces the Fe_4_O_3_ particles within the MF to realign which leads to a change of the magnetic fluid RI and consequently to a change in the resulting interference spectrum of the fiber structure. The changes in the transmission spectrum of the proposed structure then can be related to the value of the magnetic field strength.

The sensitivity of the sensor depends on the dip wavelength shift, which can be estimated by the formula^17^

8 $${\rm{\Delta }}\lambda =\frac{{\rm{\Delta }}\varphi }{2\pi }\cdot FSR=\frac{{\rm{\Delta }}{B}_{m}D}{{B}_{p}l+{B}_{m}D}\cdot \lambda $$

where Δ*ϕ* is the combined phase difference accumulated due to the birefringence of the PMF and MFC coated with MF layer, *B*_*m*_ and *B*_*p*_ are the birefringence values for the magnetic-fluid-clad-MFC and PMF respectively, Δ*B*_*m*_ is the change of the birefringence values for the magnetic-fluid-clad-MFC with the external magnetic field, *l* is the length of the PMF, *D* is the length of the coupling region of the magnetic-fluid-clad-MFC of the MF layer, which is ~3.5 mm in our experiments and *λ* is the wavelength of operation.

Magnetic field-dependent birefringence of MF (typically *B*_*m*_ ~ 1.2 × 10^−3^ for magnetic fields above 30 mT)^18^ is approximately one order of magnitude greater than that of PMF (*B*_*p*_ ~ 10^−4^). However, since the length of the magnetic-fluid-clad-MFC is D ~ 3.5 mm, which is 100 times shorter than that of PMF (*l* ~ 700 mm), in our experiment *B*_*p*_ *l* ≫ *B*_*m*_ *D*, so that

9 $${\rm{\Delta }}\lambda \approx \frac{{\rm{\Delta }}{B}_{m}D}{{B}_{p}l}\cdot \lambda $$

10 $$Sensitivity=\frac{{\rm{\Delta }}\lambda }{{\rm{\Delta }}MFS}\approx \frac{{\rm{\Delta }}{B}_{m}D}{{\rm{\Delta }}MFS\cdot {B}_{p}l}\cdot \lambda $$

where Δ*MFS* is the difference of the magnetic field strength.

**Figure 4:**
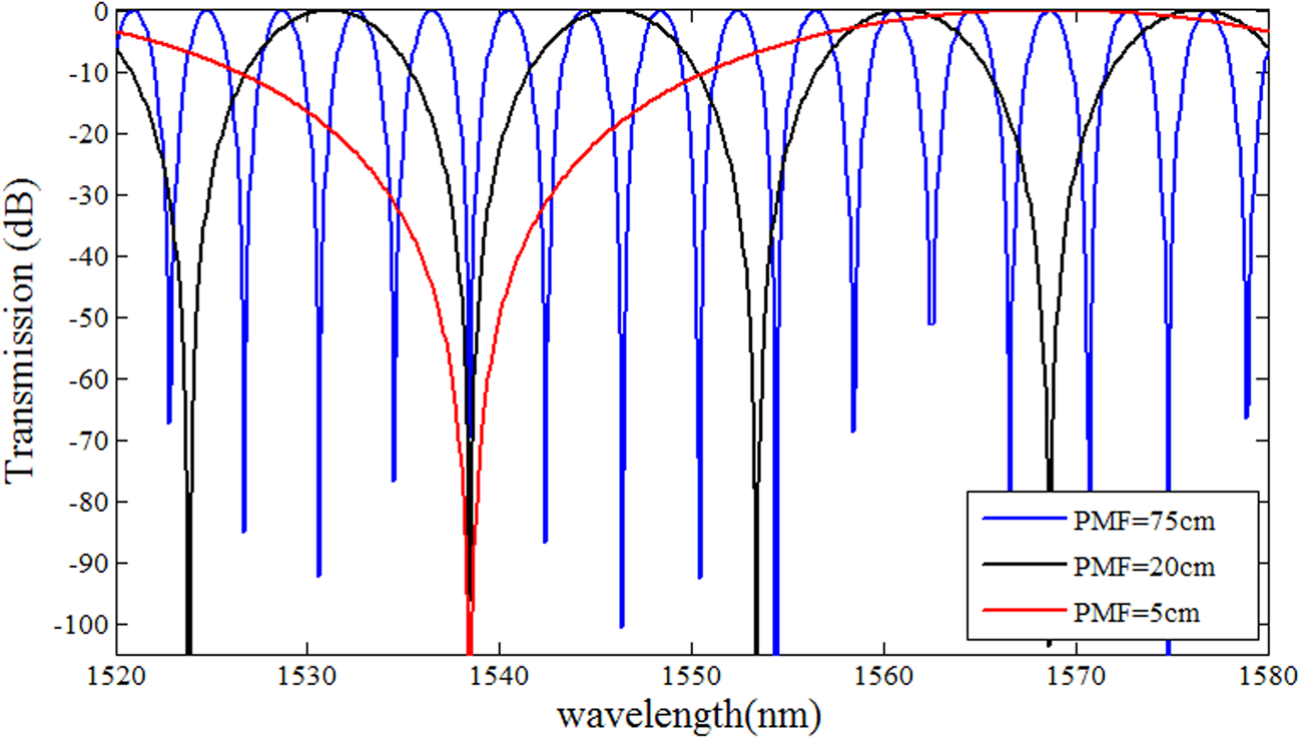
Simulated transmission of the proposed structure for different lengths of the PMF section.

Equation (10) shows that the sensitivity of the proposed sensor is in inversely proportional to the length of PMF (*l*) and directly proportional to the birefringence of the MFC (Δ*B*_*m*_). Since the birefringence of the MFC is determined by its length and the MF layer thickness both of which are fixed for a packaged MFC, the sensitivity of the structure can be enhanced by shortening the PMF section. On the other hand, since a reduction in the PMF length leads to an increase of the free spectral range of the transmission spectrum, the PMF section should be sufficiently long to ensure the presence of at least one transmission dip within the wavelength range of interest. Thus the length of PMF cannot be shortened without limitations and setting the length of the PMF will involve reaching a compromise between sensitivity and practical need to operate within the wavelength range of an available source. Figure 4 shows simulated transmission spectra for the proposed structure with three different lengths of the PMF, illustrating the corresponding changes in the FSR.

## Experimental Results and Discussion

The experimental setup for the magnetic field sensor characterization is shown in Fig. 1(a). A broadband optical source was connected to the fiber sensor and the spectral response of the sensor was detected by an optical spectrum analyzer. The magnetic field was generated by a double-tuned adjustable air gap electromagnet (DXWD-100, C-type), while the sensor head was center-aligned within the air gap in a uniform magnetic field. The electromagnet was driven by a bipolar constant-current power supply with a built-in high-precision Gauss Meter and a complete closed-loop control circuit to ensure magnetic field rapidly reaches a stable and uniform value. The air gap was fixed to 40 mm and diameter of the magnetic pole was 60 mm. The magnetic field strength linearly increased with the increase of the source current up to 200 mT. The fluctuation in the magnetic field strength was less than 1 mT, reaching stability in less than 0.5 sec.

**Figure 5:**
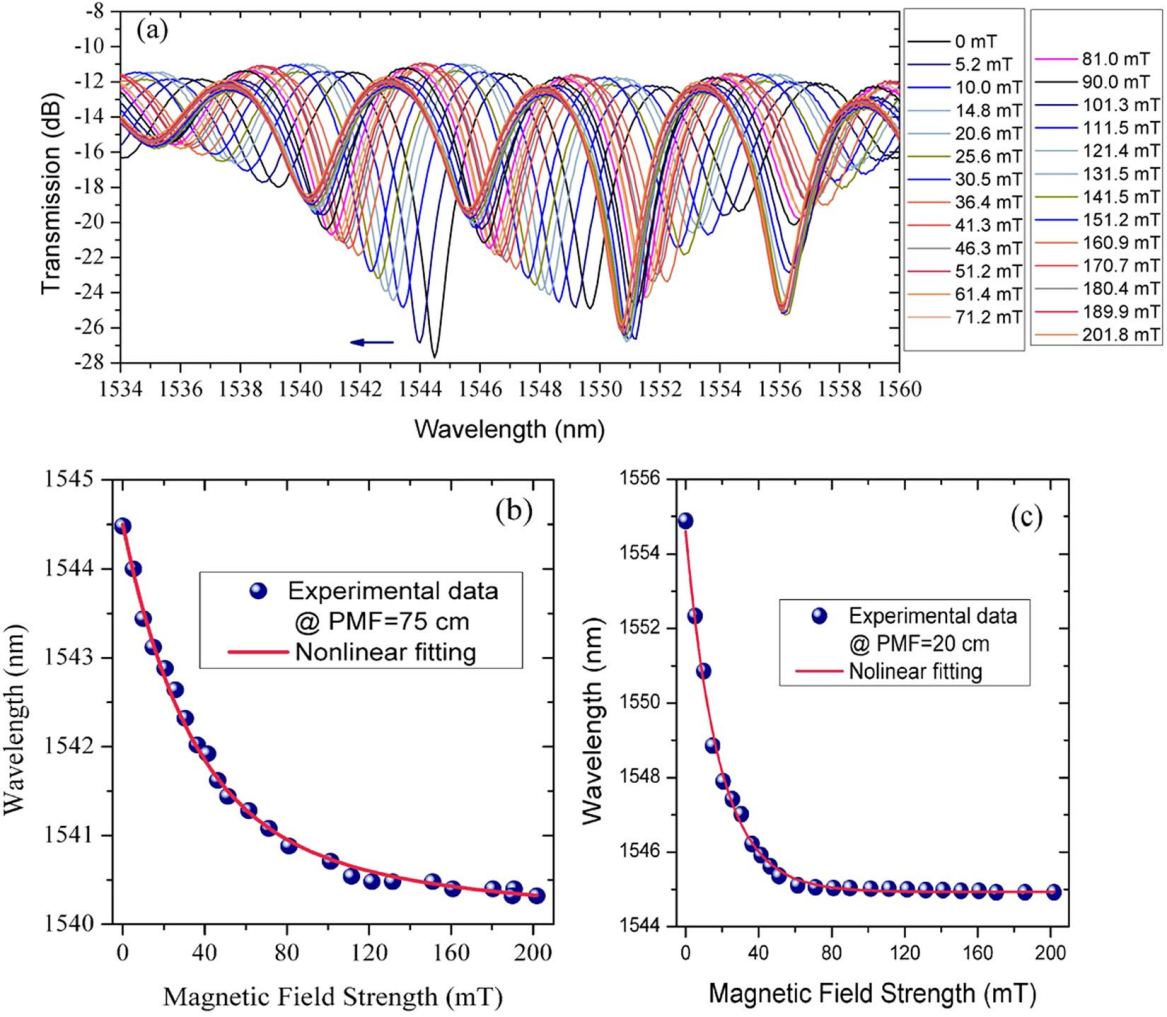
(**a**) Experimental transmission spectra of the MFC-Sagnac loop (PMF length = 75 cm) immersed in a MF with 10 nm-diameter Fe_4_O_3_ particles with a concentration of 1 mg/mol for different magnetic field strengths from 0 to 200 mT; (**b**) Selected transmission dip wavelength (*λ*_0_ = 1544.48 nm) versus magnetic field strength for the sample with a PMF length of 75 cm; (**c**) Selected transmission dip wavelength (*λ*_0_ = 1554.88 nm) for the sample with a PMF length of 20 cm versus magnetic field strength increasing from 0 to 200 mT.

Figure 5(a) shows the experimentally measured transmission spectra of the proposed sensor under different magnetic field strength values ranging from 0 to 200 mT. As predicted in the previous section, an increase in the magnetic field strength results in the shift of the transmission dip towards shorter wavelengths. Figure 5(b) is a plot of the central wavelength of the selected transmission dip (near 1544 nm) against the applied magnetic field strength. As can be seen from the plot, for the low magnetic field strength range (from 0 to 20 mT) the dip wavelength shifts rapidly from 1544.48 nm to 1542.88 nm, which is followed by a much slower shift from 1542.88 nm to 1540.5 nm in the range of fields from 20 mT to 200 mT. This is likely due to a saturation of the MF magnetization at higher magnetic field strengths when most of the nanoparticles agglomerate as magnetic chains. Above complete saturation (when all the nanoparticles are re-aligned), the RI of the MF is no longer dependent on changes in magnetic field. Figure 5(c) illustrates the dependence of the central wavelength of a transmission dip near 1554.88 nm versus applied magnetic field for the fiber structure with a PMF section length of 20 cm. The corresponding FSR is increased to 14 nm in comparison with 5 nm seen in Fig. 5(a) for the PMF length of 75 cm.

**Figure 6:**
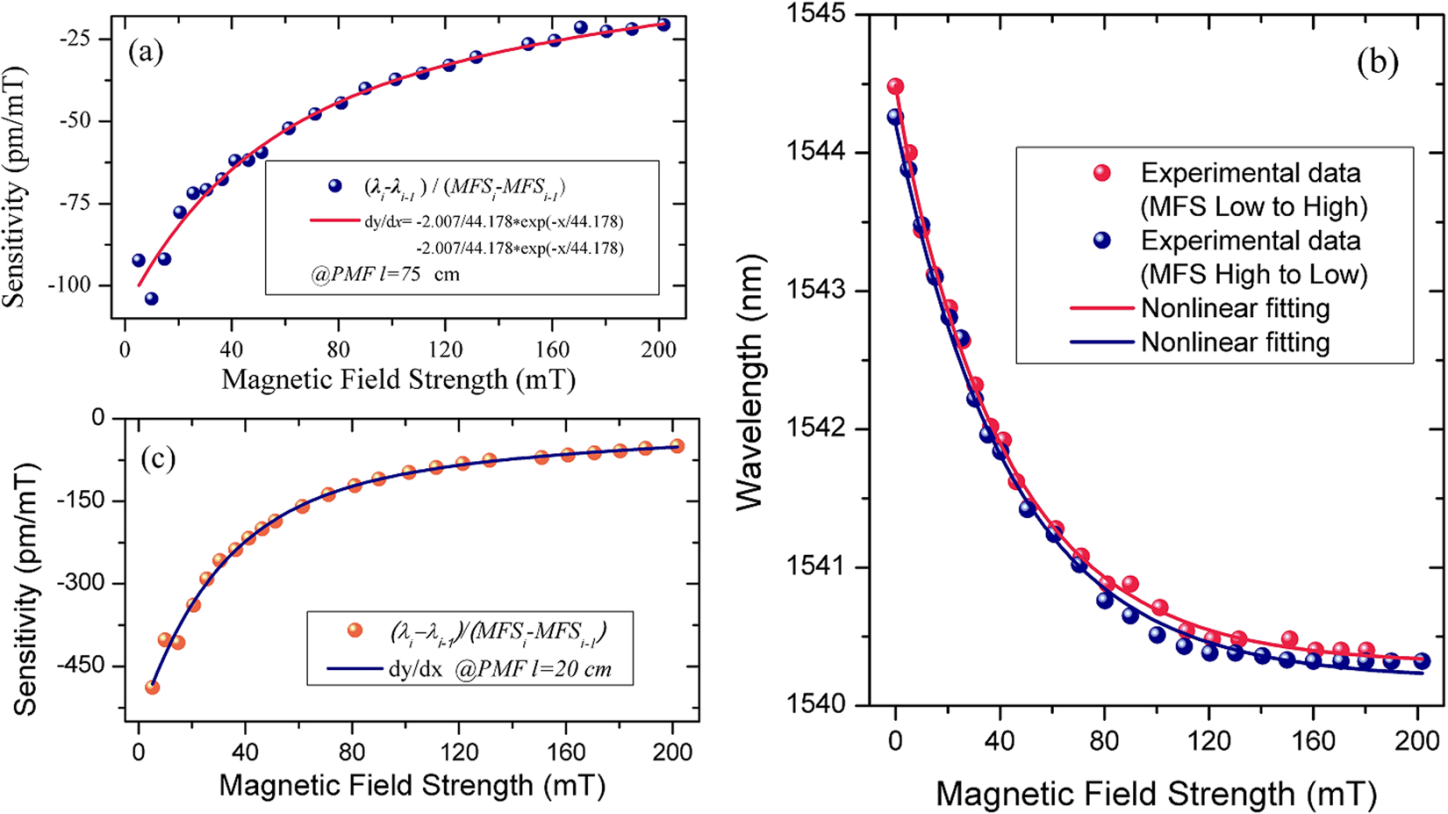
(**a**) Calculated sensitivity versus magnetic field strength in the range from 0 to 200 mT; (**b**) Measured wavelength shift of the selected transmission dip (at *λ*_0_ = 1544.48 nm, PMF length 75 cm) against increasing magnetic field from 0 to 200 mT (red) and decreasing back to 0 mT (blue); (**c**) Measured wavelength shift of the selected transmission dip (at *λ*_0_ = 1554.88 nm, PMF length 20 cm) against increasing magnetic field from 0 to 200 mT.

Figure 6(a and c) shows the dependence of the sensitivity on the magnetic field strength calculated based on the experimental data and the fitting curve in Fig. 5(b and c). The maximum sensitivity to a magnetic field achieved in the experiments is estimated to be −100 pm/mT in the range from 0 to 200 mT, for the sample with MF of IO-A10-1, and PMF length of 75 cm, which is much better than the sensitivity of 0.5 pm/Oe for a magnetic sensor using a similar MFC-Sagnac loop without PMF, reported in reference^12^ in 2015. Similarly for the sample with the PMF length of 20 cm, the maximum sensitivity is estimated as −488 pm/mT in the magnetic field range from 0 to 200 mT. As predicted by the theoretical model, a shorter length of the PMF section leads to a higher sensitivity.

The hysteresis behavior of the sensor was also studied by increasing the magnetic field strength from 0 to 200 mT and then decreasing it down to zero with the same step values while recording the wavelength shift for the selected transmission dip. The results are shown in Fig. 6(b). As one can see from the figure, while the wavelength position of the selected transmission dip does not return exactly to its initial state after the full cycle of measurements, overall the hysteresis is negligibly small.

**Figure 7:**
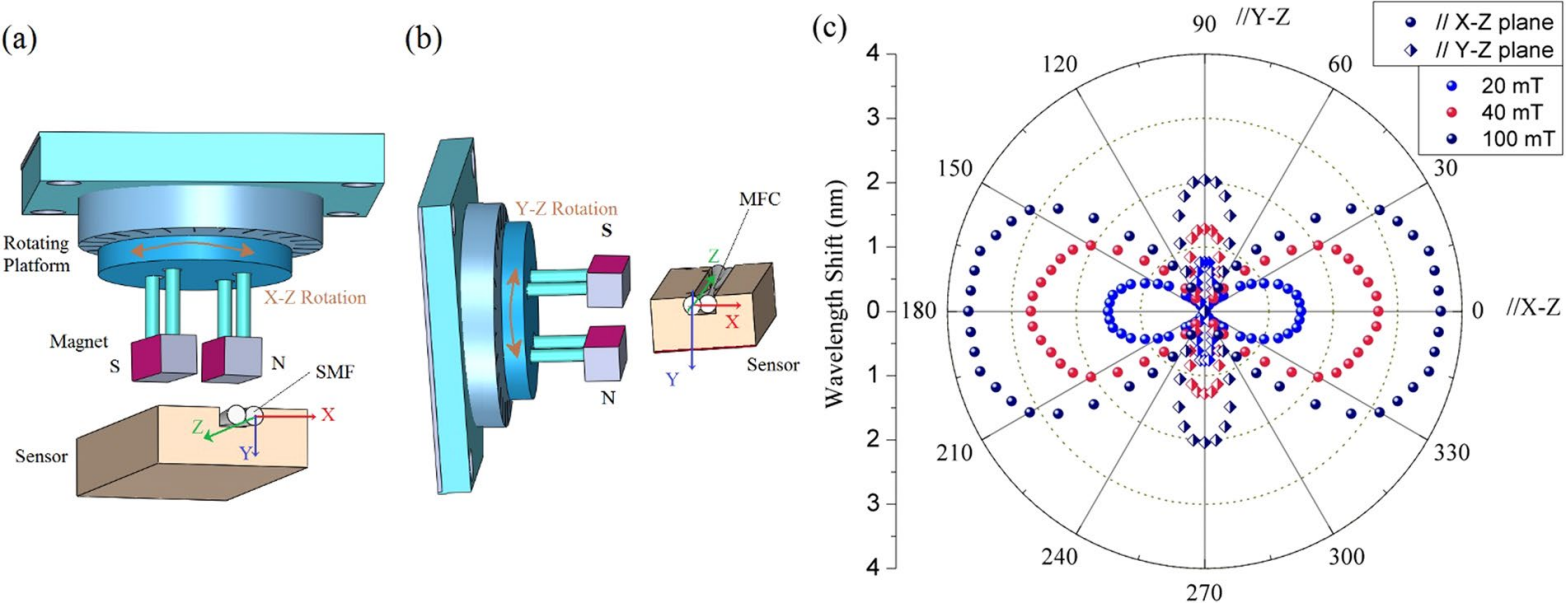
Schematic of the experimental setup for measurements of the angular field dependence in (**a**) the X-Z and (**b**) the Y-Z plane; (**c**) Experimentally measured wavelength shift from *λ*_0_ versus from 0° to 360° in the X-Z and Y-Z planes for different magnetic field strength values.

The dependence of the magnetic field orientation on the performance of the proposed sensor was also examined for the sample with the PMF length of 75 cm and the MFC waist diameter of 2.6 *μm*. For this purpose a separate experiment was set up using two permanent magnets to create a parallel and homogeneous magnetic field. A 360° rotating platform (from Thorlabs) was used to hold the two magnets positioned at a fixed distance from each other and in the vicinity of the stationary sensor head. The direction of the magnetic field applied to the sensor was changed by rotating the platform with 5° steps, in the planes X-Z and also Y-Z as shown in Fig. 7(a,b). The rotation angle *α* is defined as the clockwise angle between the normal line of the magnetic field and to the direction the MFC axis. *α* = 0° when magnetic field axis is normal to the MFC axis, and *α* = 90° when magnetic field axis is parallel to the MFC axis. For each of the experimental configurations measurements of the wavelength shift of the selected transmission dip were carried out by first setting the magnetic field strength to a certain value (20 mT, 40 mT, and 100 mT) by using different sets of permanent magnets and then changing the rotation angle *α* from 0° to 360° in 5° steps. The results of the experiment are illustrated in Fig. 7(c). The results measured at different magnetic field strengths agree well with those presented in Fig. 5(b) and demonstrate that as expected, a smaller magnetic field strength leads to a smaller wavelength shift.

In order to explain the dependence of the wavelength shift on the magnetic field rotation angle, we need to consider the re-distribution of iron nanoparticles in the MF under the action of the applied field and with respect to the fiber (MFC) axis. When the magnetic field is applied in the vicinity of the PDMS container, the nanoparticles tend to gather around the surface of the PDMS slot, forming multi-particle chains and their density around the MFC surface becomes non-homogeneous and asymmetrical with respect to the fiber axis. This in turn results in a non-homogeneous RI of the MF surrounding the fiber surface. When the magnetic field direction is rotated in the X-Z or Y-Z planes, the chains direction and nanoparticles density around the MFC rotates accordingly, affecting the RI of the MF and ultimately the sensor’s response. The maximum wavelength shift was observed when *α* was set to 0° (magnetic field axis is normal to the MFC axis) and the minimum shift was recorded when *α* was 90° (magnetic field axis is parallel to the MFC axis). This is true for both field rotation experiments in the X-Z and Y-Z planes. The maximum wavelength shift in the X-Z plane is greater than that in the Y-Z plane, which most likely a result of the shape of the MFC placed in the PDMS slot.

**Figure 8:**
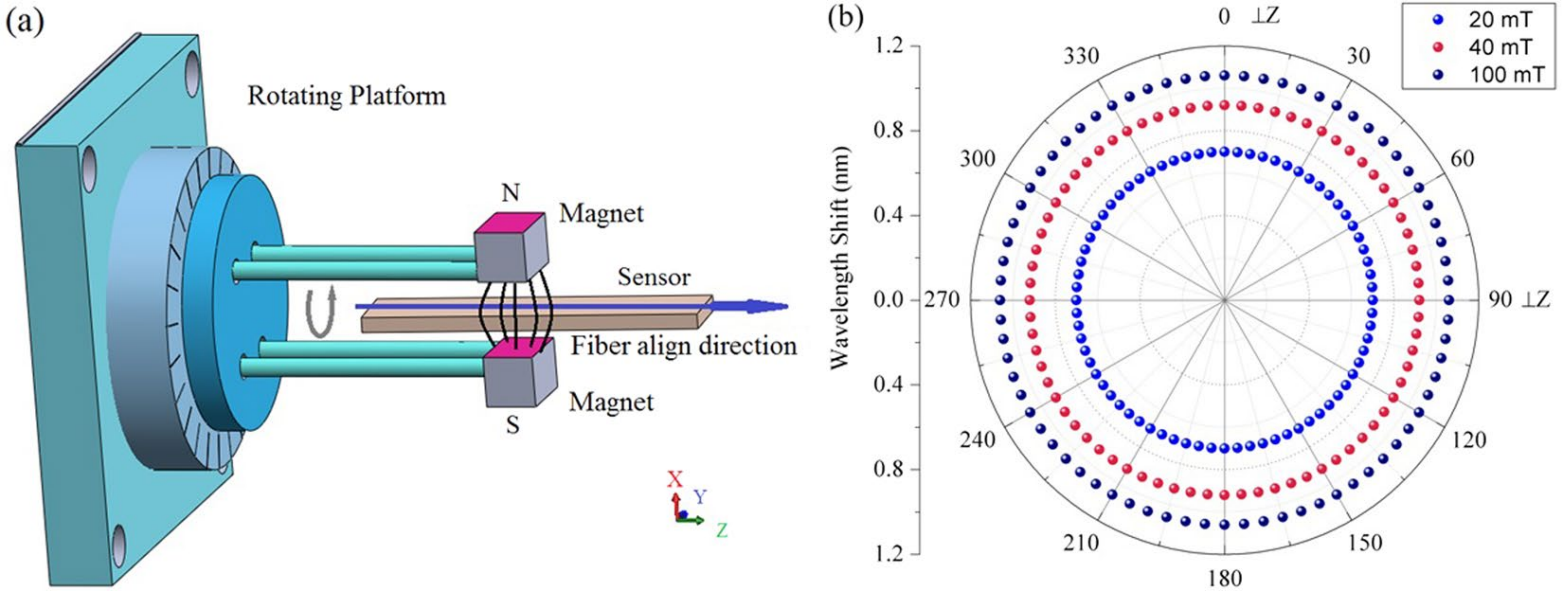
(**a**) The experiment set up for measurements of the angular filed dependence in X-Y plane; (**b**) Wavelength shift from *λ*_0_ when the rotation angle changed from 0° to 360° in the X-Y plane.

Another experiment was carried out to study the dependence of the performance of the proposed sensor on the magnetic field direction in the plane perpendicular to the MFC axis. A schematic diagram of the experiment is shown in Fig. 8(a), where the magnetic field is rotated around the Z axis. Initially when the magnets were placed near the center of the MFC, the wavelength of the transmission dip drifted slowly from its initial position towards a shorter wavelength, reaching a stable value after several minutes. This effect was observed in each of the experiments with different field strengths. We believe the initial drift was due to the fact that the magnetic field was created by permanent magnets whose cross section was smaller than the sensing length of the MFC (determined by the length of the MFC covered by the MF). Immediately after the initial application of the magnetic field the nanoparticles in the magnetic fluid moved towards the center of the MFC, causing a small change of the RI and the corresponding wavelength shift. Once the localized concentration of magnetic particles within the magnetic field cross-section becomes stable, no further changes in the RI of MF occurred when the magnetic field direction was rotated in the X-Y plane. The results of this experiment (after the transmission dip position stabilized) are shown in Fig. 8(b). It can be seen that the transmission dip wavelength does not shift as the magnetic field is rotated in the X-Y plane. This can be explained by the fact that in this configuration the magnetic field direction is perpendicular to the MFC axis at all times, and thus the particles density along the MFC is always symmetrical. When the magnetic field direction is rotated around the Z axis, the nanoparticles’ density does not change, so that the surrounding RI remains the same. The above experimental results could be used for improving the sensitivity of the proposed fiber structure and the development of a 3D-magnetic field sensor.

## Conclusions

A MF based sensor of magnetic field using a combination of a MFC and a Sagnac loop has been proposed and studied both theoretically and experimentally. When magnetic field acts upon the MFC waist section covered with the MF, the overall interference spectrum changes due to the variation in the magnetic fluid RI. The small waist diameter of the MFC leads to a large proportion of the evanescent field in contact with the magnetic fluid and this enables a stronger influence of the RI of the medium surrounding the MFC waist on the resulting interference spectrum of the structure, leading to its high magnetic field sensitivity. We have developed a simple theoretical model, allowing for the analysis of the influence of different parameters of the proposed structure on its interference spectrum. The model agrees well with the experimentally measured spectra.The sensitivity of the proposed sensor can be enhanced by shortening the length of the PMF section. The maximum magnetic field sensitivity of −488 pm/mT in the range from 0 to 200 mT has been experimentally demonstrated with a MFC of small waist diameter (~2.6 *μ*m) and PMF length of 20 cm. We studied the hysteresis behaviour of the sensor and demonstrated that the value of hysteresis is small. We also studied and analysed the influence of the magnetic field direction on the sensor’s response. This proposed sensor offers advantages for applications requiring higher sensitivity and a wide magnetic field range.

## Methods

In the manuscript, the proposed theoretical model based on the coupled-mode theory was analyzed numerically by means of Matlab software package (Mathworks).

The MFC was fabricated by simultaneously tapering and fusing two standard single-mode fibers (SMF-28) using a method known as the microheater brushing technique^19^. An MFC sample with a waist diameter ~2.6 *μ*m was used in our experiments. The PDMS container was fabricated to a cuboid with a slot cut through its center along the long side. The MFC was immobilized within the slot by securing the output fiber ends with a UV-curable glue. Two small holes in the top cover of the container are located above the ends of the slot for injecting the MF liquid and exhausting the air.

The MF sample (IO-A10-1) was employed with 10 nm Fe_4_O_3_ particles at a concentration of 1 mg/ml, which was purchased from Cytodiagnostics Inc.
